# IFN-γ and TNF-α synergism may provide a link between psoriasis and inflammatory atherogenesis

**DOI:** 10.1038/s41598-017-14365-1

**Published:** 2017-10-23

**Authors:** Nehal N. Mehta, Heather L. Teague, William R. Swindell, Yvonne Baumer, Nicole L. Ward, Xianying Xing, Brooke Baugous, Andrew Johnston, Aditya A. Joshi, Joanna Silverman, Drew H. Barnes, Liza Wolterink, Rajan P. Nair, Philip E. Stuart, Martin Playford, John J. Voorhees, Mrinal K. Sarkar, James T. Elder, Katherine Gallagher, Santhi K. Ganesh, Johann E. Gudjonsson

**Affiliations:** 1 National Heart Lung and Blood Institute, National Institutes of Health, Bethesda, MD USA; 2 Department of Dermatology, Univ. of Michigan, Ann Arbor, MI USA; 3 Department of Dermatology, Case Western Reserve University, Cleveland, OH USA; 4 Department of Surgery, Division of Vascular Surgery, Univ. of Michigan, Ann Arbor, MI USA; 5 Department of Internal Medicine, Division of Cardiovascular Medicine, and Department of Human Genetics, Univ. of Michigan, Ann Arbor, MI USA

## Abstract

Chronic inflammation is a critical component of atherogenesis, however, reliable human translational models aimed at characterizing these mechanisms are lacking. Psoriasis, a chronic inflammatory skin disease associated with increased susceptibility to atherosclerosis, provides a clinical human model that can be utilized to investigate the links between chronic inflammation and atherosclerosis development. We sought to investigate key biological processes in psoriasis skin and human vascular tissue to identify biological components that may promote atherosclerosis in chronic inflammatory conditions. Using a bioinformatics approach of human skin and vascular tissue, we determined IFN-γ and TNF-α are the dominant pro-inflammatory signals linking atherosclerosis and psoriasis. We then stimulated primary aortic endothelial cells and *ex-vivo* atherosclerotic tissue with IFN-γ and TNF-α and found they synergistically increased monocyte and T-cell chemoattractants, expression of adhesion molecules on the endothelial cell surface, and decreased endothelial barrier integrity *in vitro*, therefore increasing permeability. Our data provide strong evidence of synergism between IFN-γ and TNF- α in inflammatory atherogenesis and provide rationale for dual cytokine antagonism in future studies.

## Introduction

Psoriasis is a common chronic inflammatory and hyper-proliferative skin disease that affects 2–3% of the US population and is associated with significant metabolic and cardiovascular co-morbidities^1–3^. Several epidemiologic studies^4^ demonstrate an association between psoriasis and cardiovascular diseases independent of traditional cardiovascular risk factors^1,2^. Strikingly, this association is strongest in younger patients with severe cutaneous disease^4^, with a major consequence of this being premature death compared to patients without psoriasis due to atherosclerotic cardiovascular disease^5^. Several lines of evidence point towards a major role for skin-derived inflammatory mediators in the promotion of atherogenesis. This includes the striking paucity of overlap between psoriasis and atherosclerosis genetic risk loci^6,7^ making shared genetic predisposition unlikely. Furthermore, mouse models of psoriasis, such as the KC-Tie2 mouse model^8^, where transgene expression is confined to keratinocytes of the epidermis, have increased and accelerated atheroma formation^9^. However, the mechanistic process driving an increased susceptibility to atherosclerosis from skin inflammation in human psoriasis remains poorly understood. In this manuscript we sought to identify potential pathways linking inflammatory atherogenesis to open the door for future mechanistic investigations and treatment strategies to decrease CVD in psoriasis.

Psoriasis and atherosclerosis share many similar underlying inflammatory mechanisms. In psoriasis, inflammation at the dermal-epidermal junction drives keratinocyte proliferation and development of psoriatic plaque. In atherosclerosis, inflammatory cells accumulate at sites of endothelial injury, contribute to atherosclerotic plaque, and mediate plaque instability leading to myocardial infarction^10^. We previously showed that pathways involved in atherosclerosis are activated in skin lesions from psoriatic skin, and protein products of these pathways are elevated in the blood^11^. Beyond this, similarities between these two processes have largely remained unexplored.

To address the link between these diseases we used a systems biology analyses to identify critical pathways shared between psoriasis and atherosclerosis. Based on transcriptome analysis, we identified IFN-γ and TNF-α as the two top inflammatory mediators shared between the two disease processes. Importantly, we show that both IFN-γ and TNF-α are increased in the serum of patients with moderate-to-severe psoriasis and that their respective receptors in atherosclerotic plaques are increased. We demonstrate that these cytokines amplify inflammatory responses in atherosclerotic blood vessels and human aortic endothelial cells with the role of TNF-α in this process being primarily through amplification of Th1 responses. Stimulation of primary aortic endothelial cells and *ex-vivo* atherosclerotic tissue with IFN-γ and TNF-α synergistically increased monocyte and T-cell chemoattractants and adhesion molecules, concomitant with a decrease in endothelial barrier integrity. Our results suggest IFN-γ/TNF-α synergy may provide a critical pro-inflammatory link between psoriatic skin inflammation and distant vessel atherosclerosis, and identifies that potential blockade of both IFN-γ and TNF-α as a novel therapeutic target in psoriasis-associated atherogenesis.

## Results

### Psoriasis and atherosclerosis exhibit significant overlap of their transcriptomes

**Figure 1:**
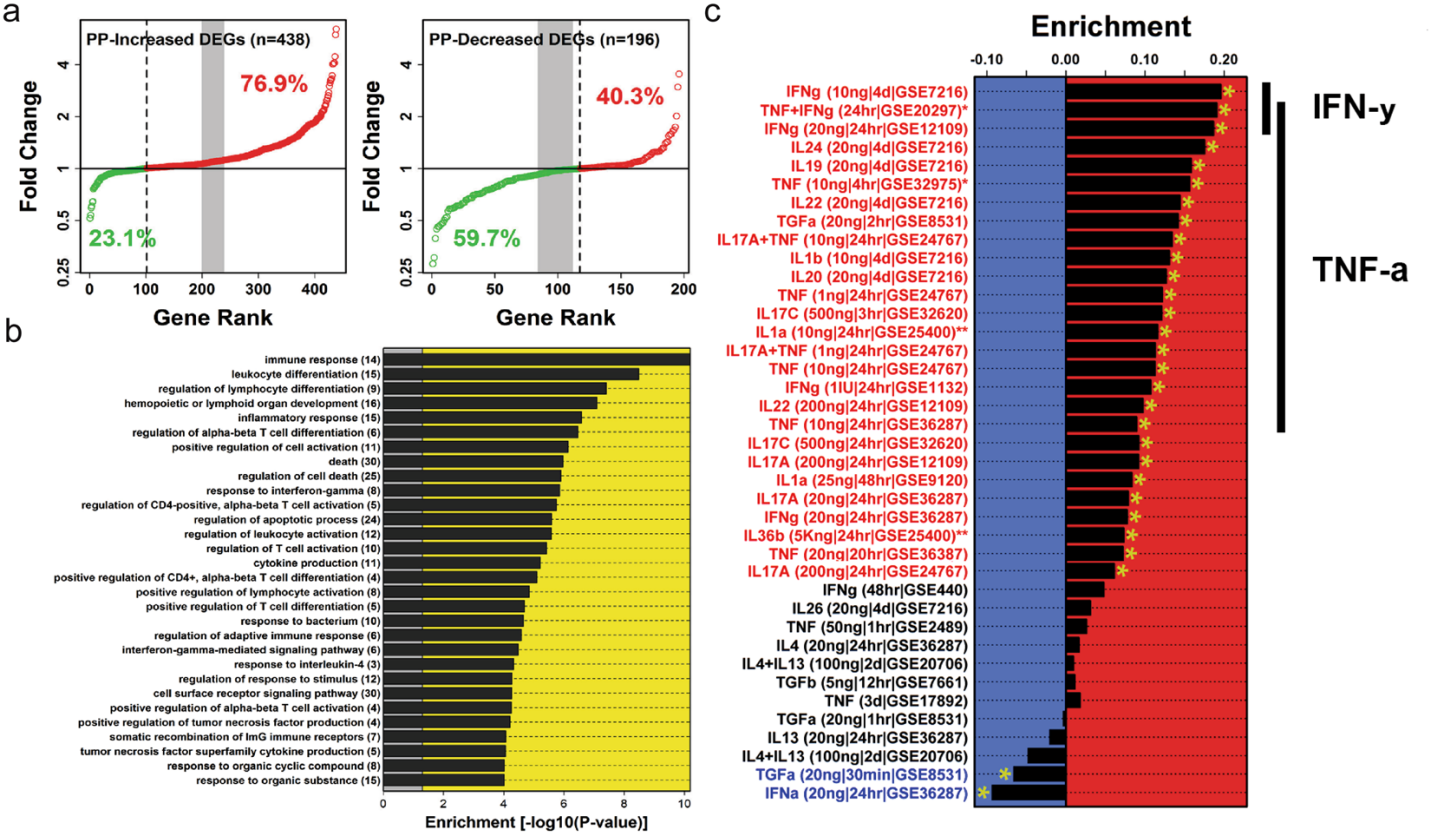
Psoriasis and atherosclerosis have overlapping gene expression and biologic processes. (**a**) Using a systems biology approach, we compared global transcriptome datasets of psoriasis skin (n = 216 to post-mortem samples of carotid atherosclerotic plaque (n = 13 early stage; n = 16 advanced stage) and determined 76.9% of increased DEGs and 59.7% of decreased DEGs are observed in both tissues. (**b**) Further analysis utilizing Gene Ontology biological process categories demonstrated that 66 Gene Ontology biological processes are enriched in both psoriatic and advanced atherosclerotic plaque (top 30 shown, p < 0.0001), and correspond to immunological processes such as inflammatory response, response to IFN-γ, regulation of T cell activation and antimicrobial responses. The number of increased genes associated with each GO term is listed in parentheses. (**c**) Keratinocytes were then systematically stimulated with combinations of cytokines enriched in both psoriasis and atherosclerotic lesions determined by RNAseq. Positive statistics denote enrichment for genes induced by a given cytokine treatment, while negative statistics denote enrichment for genes repressed by a given cytokine treatment. Black bars on left highlight IFN-γ and TNF enriched signatures.

We hypothesized that there is significant overlap in the differentially expressed genes (DEGs) of lesional psoriasis skin and atherosclerotic plaques. Using our previously described systems biology approach^12^, we tested this hypothesis by comparing global transcriptome datasets of psoriasis skin (n = 216) to post-mortem samples of carotid atherosclerotic plaque (n = 13 early stage; n = 16 advanced stage) to elucidate the overlapping DEGs^12^. Of the 438 genes increased in psoriasis (FC > 2; FDR < 0.05), 76.9% were also increased in advanced compared with early stage atherosclerotic plaques (P = 1.71 × 10^−35^) (Fig. 1a). Additionally, 59.7% of the 196 psoriasis-decreased genes were also decreased in advanced atherosclerotic plaque (P = 0.008) (Fig. 1a). We then determined the biological processes associated with these shared DEGs utilizing a Gene Ontology (GO) biological process approach. Genes increased in both psoriatic and advanced atherosclerotic plaque were enriched with respect to 66 GO biological process categories (P < 0.001); the majority belonging to immunological processes including inflammatory response, response to IFN-γ, regulation of T-cell activation and antimicrobial responses (Fig. 1b). GO analysis of genes decreased in psoriatic skin and advanced atherosclerotic plaques revealed enrichment for developmental and metabolic processes (data not shown). Next, we sought to determine the cytokines most likely contributing to the increased expression of the DEGs *in vivo*. To identify this, we used microarray data to perform gene-set enrichment analyses from cytokine stimulated keratinocytes, as previously described by our group^13^, and cross-analyzed this against the gene set shared between atherosclerosis and psoriasis. Strikingly, the common psoriasis/atherosclerosis gene set was disproportionately induced by IFN-γ (FDR = 1.47 × 10^−11^) and TNF-α (FDR < 0.01) (Fig. 1c). Less prominent were signatures for IL-17 and IL-20 family members (IL-19/IL-20/IL-24) (FDR < 0.05) (Fig. 1c). These analyses support that IFN-γ and TNF-α strongly contribute to the link between active psoriasis and atherosclerosis.

### Expression of IFN-γ and TNF-α receptors are increased in atherosclerotic lesions

**Figure 2:**
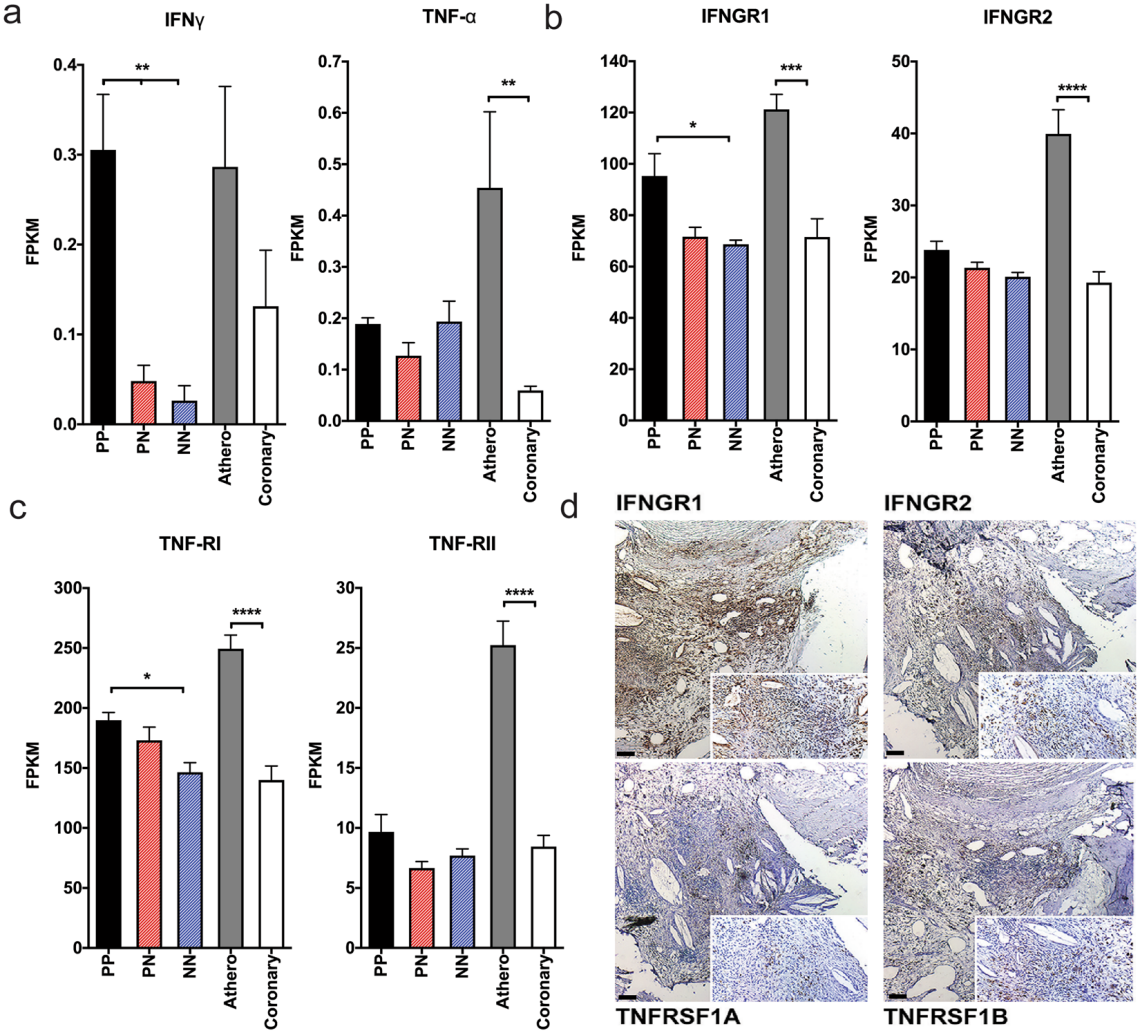
TNF and IFN-γ receptors have increased expression in atherosclerosis compared to psoriasis. (**a**,**b**,**c**) RNAseq revealed mRNA expression of the IFN-γ is elevated in psoriasis and atherosclerotic lesions, however the IFN-γ receptors (IFNGR1, IFNGR2) TNF-α and the TNF-α receptors (TNF-RI and TNF-RII) are increased in atherosclerosis lesions compared to psoriasis, (data obtained from RNA seq and values shown as FPKM (Fragments per Kilobase of transcripts per Million mapped reads). (n = 6 lesional (PP), non-lesional (PN) and healthy (NN), n = 4 atherosclerotic (athero), n = 4 healthy vascular tissue (coronary), **p < 0.01, ***p < 0.001, two-tailed Student’s t-tests). (**d**) Immunohistochemistry staining of atherosclerotic lesions for IFNGR1, IFNGR2, TNF-RI and TNF-RII confirms protein expression. Data are shown as mean ± SEM with 95% CI.

We next analyzed the mRNA expression of IFN-γ and TNF-α and their receptors in psoriasis skin and atherosclerotic tissue. We determined mRNA expression of the IFN-γ is elevated in lesional psoriasis skin compared to non-lesional and healthy skin, whereas TNF-α is elevated in atherosclerotic lesions relative to healthy coronaries. Their receptor chains *IFNGR1* and *IFNGR*2, and *TNFRSF1A* (TNF-RI) and *TNFRSF1B* (TNF-RII) were increased in coronary atheromas (1.7-, 2.1-, 1.8- and 3-fold respectively, p < 0.01 for *IFNGR* receptors, and p < 0.001 for *TNFR1* and *2*) compared to healthy coronary vascular tissue (Fig. 2b,c). In addition, *IFNGR1* and *TNFR1* receptors had significantly higher expression in lesional skin compared to healthy skin (Fig. 2b,c). To link these findings and ensure the presence of IFN-γ and TNF-α receptors in atherosclerotic tissue, we utilized immunohistochemistry, finding prominent staining of IFNGR1, IFNGR2 and TNFRSF1A (TNFR1) and TNFRSF1B (TNFR2) (Fig. 2d). Expression of IL-17 cytokines and the IL-17 associated chemokine CCL20 was higher in psoriatic skin compared to atherosclerotic tissue, whereas IL17R expression was similar between skin and atherosclerotic tissue (Supplemental Fig. 1).

### IFN-γ and TNF-α are elevated in the serum of patients with psoriasis and are associated with systemic inflammatory response

**Figure 3:**
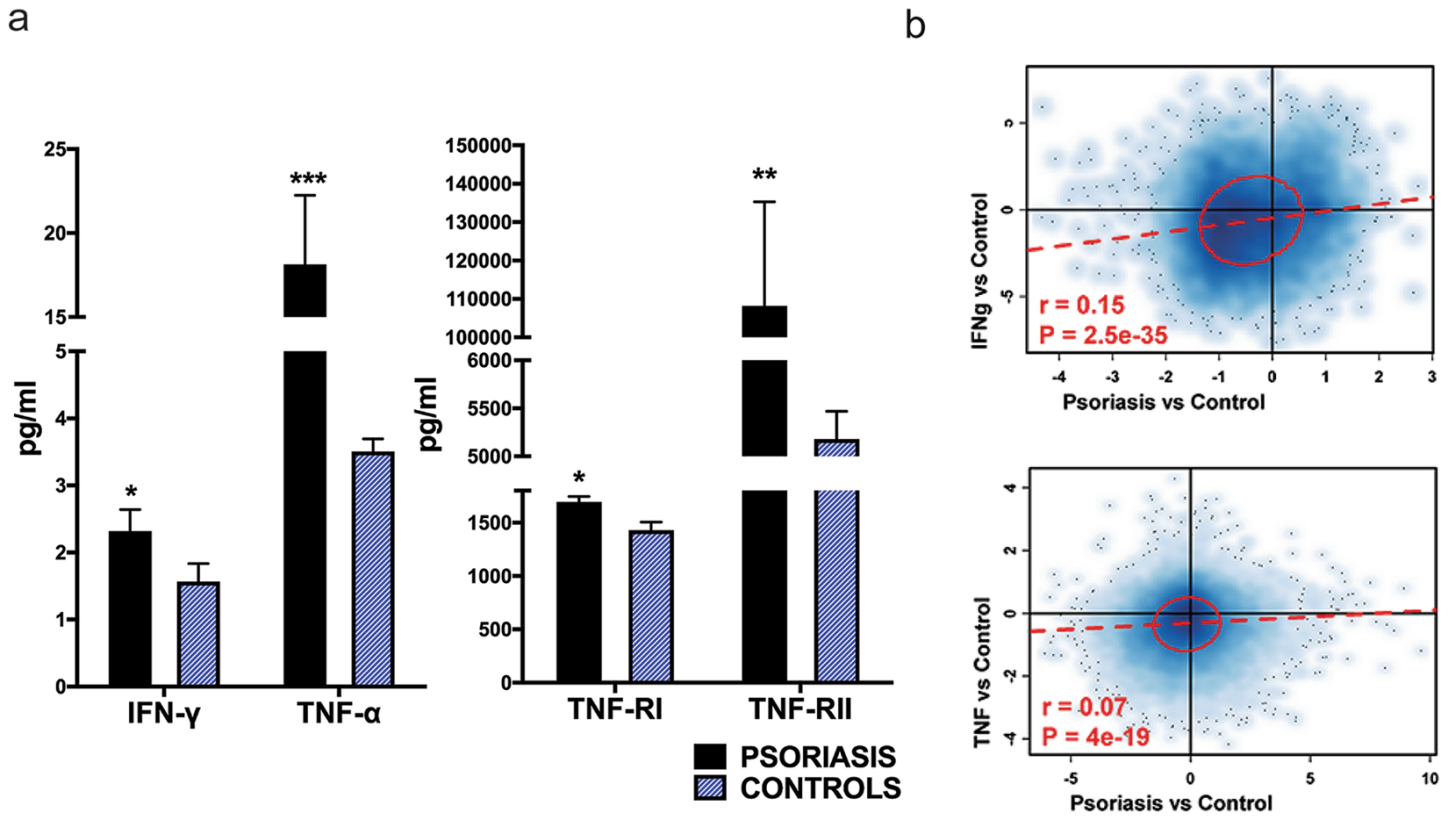
Serum IFN-γ and TNF is increased in psoriasis compared to healthy controls. (**a**) Serum IFN-γ was approximately 2.4-fold higher in patients with psoriasis compared to healthy controls, whereas serum TNF-α was about 31-fold higher (n = 112 patients, n = 54 controls, *p < 0.05, ***p < 0.001, Student’s t-tests). Serum levels of TNFRI and TNFR-II were 1.2-fold and 21-fold higher respectively in patients with psoriasis compared to controls (n = 120 patients, n = 29 controls, *p < 0.05, **p < 0.01, Student’s t-tests). (**b**) Global gene expression from PBMCs from psoriasis patients vs. control (n = 5 psoriasis vs. n = 5 controls) compared against data obtained from TNF and IFN-γ stimulated vs. unstimulated PBMCs, demonstrates positive correlation with IFN-γ and TNF-α responses (p = 2.5 × 10^−35^, p = 4 × 10^−19^ respectively, Spearman rank correlation). Data are shown as mean ± SEM with 95% CI.

We measured circulating TNF-α and IFN-γ in serum of patients with psoriasis against healthy controls. TNF-α was increased 5-fold (p < 0.01), whereas IFN-γ was increased 1.5-fold (p < 0.05) (Fig. 3a). Concomitant with elevated IFN-γ and TNF-α levels in blood of patients with psoriasis, TNFRI and TNFR-II were 1.2-fold and 21-fold higher in psoriasis compared to controls (p < 0.05 and p < 0.01) (Fig. 3a). To address whether this is associated with systemic inflammatory response we performed gene-set enrichment analysis on global gene expression data from PBMCs obtained from psoriasis patients compared against TNF and IFN-γ stimulated healthy control PBMCs. This demonstrated enriched IFN-γ and TNF-α responses in psoriatic PBMCs (p = 2.5 × 10^−35^, p = 4 × 10^−19^ respectively, Spearman rank correlation) (Fig. 3b).

### IFN-γ and TNF-α elicit synergistic inflammatory responses and atherosclerotic tissue

**Figure 4:**
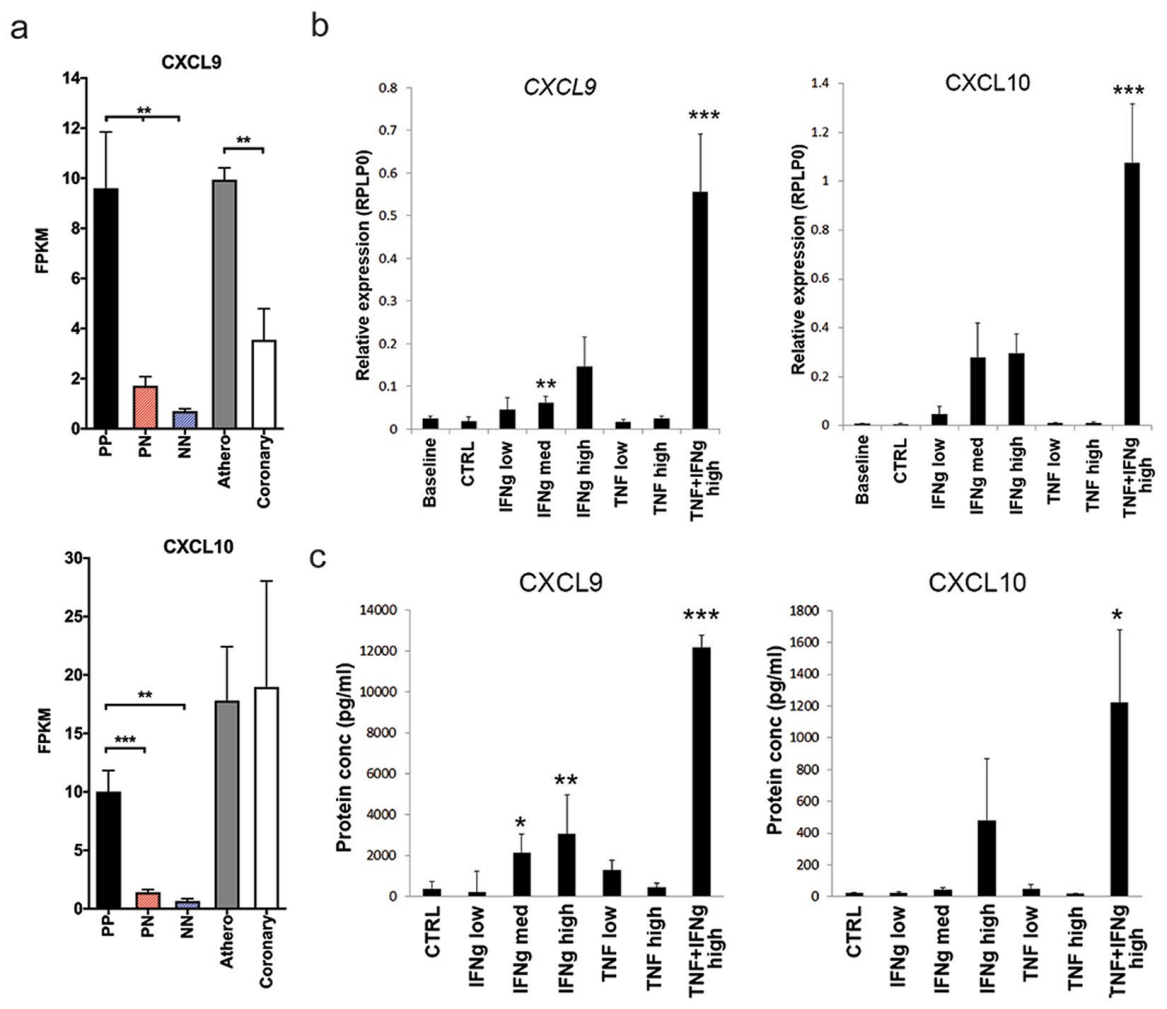
IFN-γ and TNF-α have synergistic activity on human atherosclerotic tissue *ex vivo*. (**a**) CXCL9 and CXCL10 mRNA expression in lesional (PP), non-lesional (PN) and healthy skin (NN), atherosclerotic (athero), healthy vascular tissue (coronary) obtained through RNA-sequencing and shown as FPKM values. (**b**,**c**) Atherosclerotic arterial tissue obtained post-amputation was dissected into 3 mm slices and stimulated with a high or low dose of IFN-γ or TNF-α or a combination of both for 6 hours for (**b**) mRNA expression or 72 hours for (**c**) protein secretion^6^. Dose-dependent increases in the mRNA expression and protein secretion of CXCL9 and CXCL10 were observed with IFN-γ (0.5, 5 and 50 ng/ml), whereas minimal change in expression was seen with TNF-α stimulation (1 ng/ml or and 10 ng/ml). In contrast, a striking synergistic effect was seen with IFN-γ and TNF-α stimulation (IFN-γ 50 ng/ml, TNF-α at 10 ng/ml). Data are shown as mean ± SEM with 95% CI.

We next determined CXCL9 and CXCL10, macrophage and T-cell chemoattractants, were elevated in either atherosclerotic tissue compared to healthy tissue or lesional psoriatic skin compared to non-lesional (Fig. 4a). We found a combination of IFN-γ and TNF-α stimulation of *ex-vivo* atherosclerotic tissue resulted in a marked mRNA induction and protein secretion of both CXCL9 and CXCL10 (Fig. 4b,c). These results demonstrate the ability of IFN-γ and TNF-α to elicit a marked synergistic pro-inflammatory response in endothelial cells and in atherosclerotic tissues; leading to significant increases in monocyte and T-cell chemoattractants.

### IFN-γ and TNF-α synergistic increases inflammatory responses in endothelial cells

**Figure 5:**
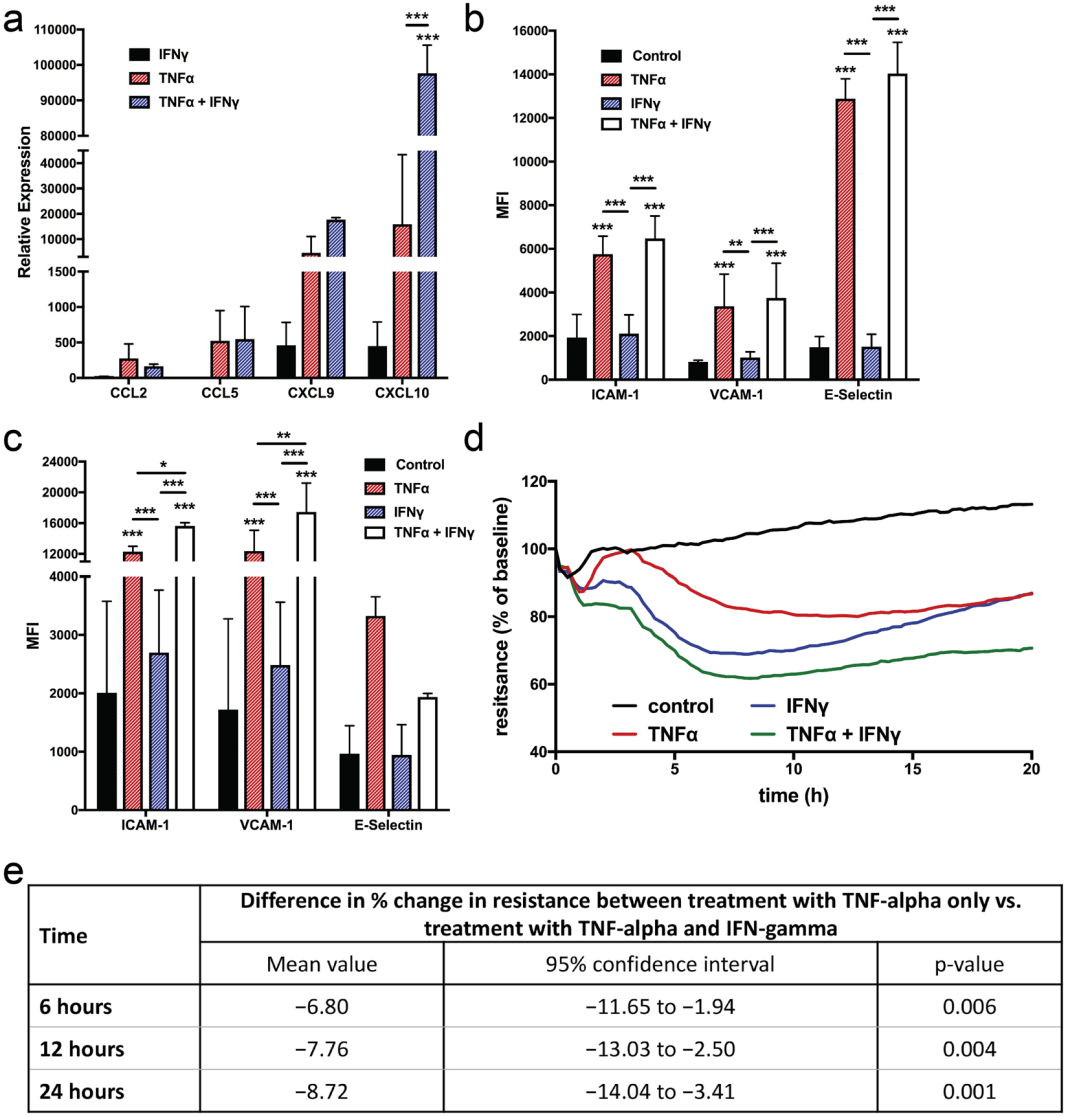
IFN-γ and TNF-α synergistically increase expression of endothelial cell activation markers *in vitro* and *in vivo*. Human aortic endothelial cells (HAoECs) stimulated with IFN-γ, TNF-α or a combination demonstrate increased (**a**) mRNA expression following 6 hours of stimulation and surface expression (shown as Mean Fluorescent Intensity (MFI)) of endothelial cell activation markers following (**b**) 4 and (**c**) 24 hours of stimulation, confirming a synergistic effect between IFN-γ and TNF-α (*p < 0.05, **p < 0.001, ***p < 0.0001, two-way ANOVA). (**d**,**e**) HAoEC barrier function meausred by ECIS demostrates a decrease in resistance of HAoECs following 6, 12 and 20 hours of IFN-γ and TNF-α treatment p < 0.01, WSANOVA). Data are shown as mean ± SD with 95% CI.

**Figure 6:**
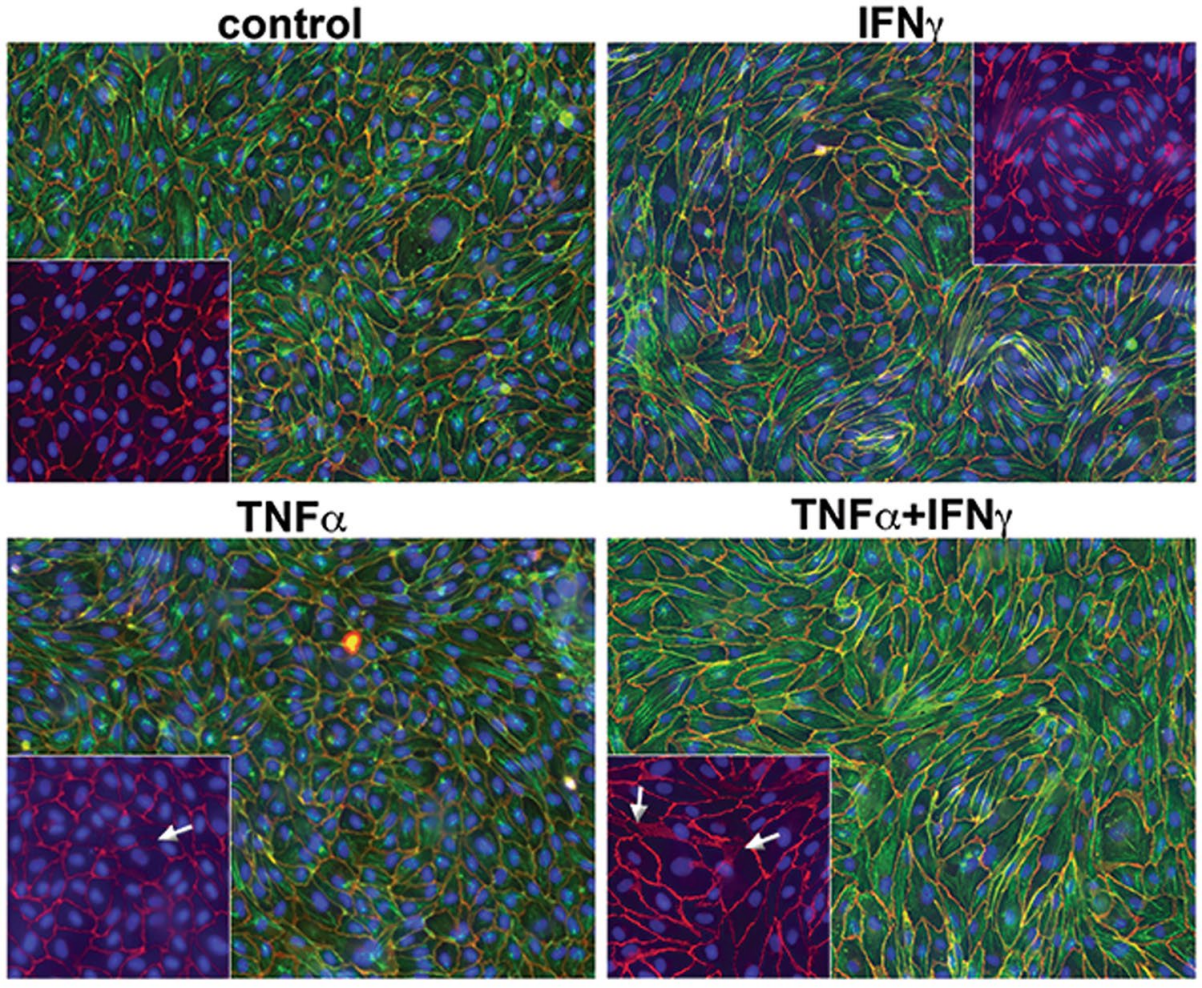
Disruption of cell-cell junction in endothelial cells with IFN-γ and TNF treatment. (**a**) Immuno-fluorescence staining of adherence junction protein VE-cadherin confirms a disruption in HAoEC cell-cell junction upon IFN-γ and TNF-α treatment.

To explore the contributions of IFN-γ and TNF-α to coronary plaque burden, vascular inflammation and atherogenesis, we stimulated human aortic endothelial cells (HAoECs) with IFN-γ, TNF-α, or both. Synergistic increases in CXCL10 (Fig. 5a) were observed following stimulation of HAoECs with IFN-γ and TNF-α compared to unstimulated cultures. Whereas IFN-γ increased the expression of CXCL9 and CXCL10 by about 600-fold (both) and TNF-α by about 13 and 60-fold, respectively, combination of IFN-γ and TNF-α induced mRNA expression of CXCL10 by approximately 95,000-fold respectively compared to unstimulated cells (p < 0.001, n = 3). In contrast the vascular adhesions molecules ICAM-1, VCAM-1 and E-selectin were induced primarily by TNF-α or IFN-γ + TNF-α (Fig. 5b,c). We then tested the integrity of the endothelial barrier function over an incubation period of 20 hours. IFN-γ and IFN-γ + TNF-α decreased endothelial cell resistance as early as 6 hours. Although IFN-γ and TNF-α individual treatments induced resistance at 6 and 12 hours, HAoECs began to recover back to control levels (Fig. 5d,e). Notably, the combination of IFN-γ + TNF-α inhibited recovery, suggesting the combination of these cytokines damages endothelial cell barrier, increasing permeability (Fig. 5d,e). This finding was confirmed by immunohistochemistry, where IFN-γ + TNF-α disrupt the endothelial junction visualized by VE-cadherin staining (Fig. 6a). Thus, these results demonstrate the ability of IFN-γ and TNF-α to elicit a synergistic pro-inflammatory response in endothelial cells and in atherosclerotic tissues; leading to significant increases in monocyte and T-cell chemoattractants and inflammatory adhesion molecules, and support that IFN-γ and TNF-α are the principal link between active psoriasis and atherosclerosis.

## Disscussion

Multiple epidemiological studies^1,4^, but not all^14,15^, have suggested association between psoriasis and cardiovascular disease. Here we provide both *in vitro* data and unbiased bioinformatic analyses to provide evidence for the involvement of two pro-inflammatory cytokines, IFN-γ and TNF-α, as key factors connecting inflammation in psoriasis and atherosclerosis. Our results are consistent with the hypothesis that increased IFN-γ and TNF-α in psoriasis is associated with a systemic pro-inflammatory gradient from inflamed skin, which then promotes inflammatory responses in both aortic endothelial cells and atherosclerotic tissue in a synergistic manner. We found limited evidence for involvement of other cytokines including IL-17 in this process, and IL-17 mRNA expression or IL-17 responses were not prominent in atherosclerotic tissues (Suppl. Figure 1). Indeed, IL-17 has received both putative and noncontributory roles in atherosclerosis. Thus, low serum levels of IL-17A have been associated with a higher risk of major cardiovascular events in Caucasian patients with acute MI^16^, and in a murine model of myocardial infarction, IL-17A promoted stabilization of atherosclerotic plaques through an interleukin-17-dependent pathway^17^. Therefore, more work is necessary to better define IL-17 role in atherosclerosis. While it is possible, and even likely, that other pathways not interrogated in our analyses contribute to this association, our data is supportive of dominance of Th1 responses in atherosclerosis and indicate that the role of TNF-α in this process may be primarily through amplification of Th1 responses involving IFN- γ. Our findings provide dual targets as a therapeutic regime to decrease the risk of myocardial infarction in psoriasis patients^18^ and provide a biologic mechanism for an epidemiologic association established through multiple prior studies^1,2^. Our study findings support the notion of potential use of both IFN- γ and TNF-α in ameliorating inflammatory atherogenesis.

The role of IFN-γ in the pathogenesis of psoriasis is not fully clear. Whereas the roles of TNF-α and IL-17 are critical in maintaining disease activity in psoriatic skin, mostly through clinical trials showing high therapeutic efficacy in targeting these two cytokines^19–22^, similar evidence for the importance of IFN-γ is lacking^23^. IFN-γ is a prominent cytokine in psoriatic lesions and intradermal injection of IFN-γ induces a range of pro-inflammatory cytokines and chemokines including the Th1 chemokines CXCL9 and CXCL10, and the dendritic cell chemokines CCL2 and CX3CL1^24^, chemokines that are a prominently expressed in psoriasis^25^. Importantly, IFN-γ and activation of downstream signaling of IFN-γ is shown to correlate with disease severity in uninvolved skin of psoriatic patients and to parallel T-cell and dendritic cell infiltration in skin^24,26^. In this context, it is of interest that disease severity in psoriasis was recently shown by our group to be associated with the degree of aortic vascular inflammation detected by FDG PET/CT^27^. Taken together, these data along with our own data showing heightened IFN-γ response in blood (Fig. 3) suggests that IFN-γ may be a key mediator of the systemic effect of psoriasis inflammation. The potential role of IFN-γ in atherosclerotic plaque development is supported by evidence from mouse models of plaque formation, including the ApoE and LDLR KO mice^28,29^, showing serologic neutralization or genetic absence of IFN-γ significantly reduces atheroma formation^30,31^. Similarly, STAT1 deficiency, a signal transduction pathway downstream of IFN-γ^32^, reduces atherosclerotic lesions, foam cell formation, and macrophage apoptosis^32,33^. However, no studies on the efficacy of anti-IFN-γ treatment on atherosclerosis have been performed.

Psoriasis and atherosclerosis share several potentially similar underlying inflammatory mechanisms^10^. Similar to psoriasis, atherosclerosis has a prominent Th1 signature^34,35^, with most reports on the role of IFN-γ in atherosclerosis depicting this cytokine as pro-inflammatory, having a role in the initial development and progression of atheromatous plaques. IFN-γ is shown to contribute to early endothelial dysfunction found in atherosclerosis^36^, causing the activation and differentiation of T-cells, as well as macrophage-mediated release of inflammatory cytokines, specifically TNF-α.

Of note is that other chronic inflammatory conditions, characterized by Th1 type inflammation, including rheumatoid arthritis^37^ and systemic lupus erythematosus^38,39^, are shown to be independent risk factors for acute myocardial infarction and severity of coronary artery disease manifested as multi-vessel disease^40,41^. Furthermore, disease activity in these conditions correlates with increased risk of cardiovascular death, even when controlling for traditional cardiovascular risk factors and comorbidities^41^. This association has been proposed to relate to underlying systemic inflammation^42^. Other chronic inflammatory conditions including eczema, which is initially characterized by Th2 inflammation but then progressively develops stronger Th1 responses with increased chronicity, is also associated with increased cardiovascular risk^43^. Thus, chronic inflammatory diseases contribute to increased risk of atherosclerosis and cardiovascular death, with disease severity being a key factor^1,26,27^. Interestingly, this association is not seen in asthma^44^, suggesting that the composition of the inflammatory response is important since not all chronic inflammatory states are equivalent in promoting and accelerating atherosclerosis.

The results presented in this manuscript have implications potentially beyond psoriasis alone in regards to the mechanisms involved in triggering and accelerating development of atherosclerosis. It provides a novel approach in which related or co-morbid diseases can be analyzed. While our data does not address whether skin derived IFN-γ is sufficient or necessary to trigger the development of atherosclerosis, data from animal models of psoriasis are strongly suggestive of this possibility^8^. Our data supports this scenario, and demonstrates that exogenous IFN-γ along with TNF-α is a “lethal” combination, massively upregulating inflammatory responses in both vascular endothelium and established atherosclerotic lesions. Consistent with this scenario, anti-TNF therapy has been shown to improve endothelial function and arterial stiffness in patients with moderate to severe psoriasis^45^, as well as coronary microvascular function^46^. No studies have yet addressed the role of anti-IFN-γ on vascular function in patients with psoriasis. Although we do not address other potential mechanisms, such as inflammation driven alterations of lipid, or insulin metabolism and insulin resistance^47^, this work identifies IFN-γ as a potential therapeutic target in psoriasis associated atherogenesis, and provides exciting avenues for further dissecting the shared pathogenic mechanisms between psoriasis and atherosclerosis, including the nature of the IFN-γ and TNF-α synergy, and sheds light on critical inflammatory mechanisms by which other chronic inflammatory conditions may affect, induce and accelerate atherosclerosis.

There are important limitations of our study. First, the small samples size of the human tissue transcriptomics (n = 13–16) and endothelial cell resistance (n = 5) may limit generalizability of the data. Our characterization of atherosclerotic plaque based on skin and vascular transcriptomic data is the first effort of its kind and lends support to future efforts of this kind to discover novel pathways linking inflammation to CVD.

In conclusion, our study demonstrates significant overlap between the transcriptomes of atherosclerotic plaque and psoriatic skin, identifies key common cellular and cytokine responses involved in both diseases, provides insights into the mechanisms by which cutaneous inflammation may feed into and accelerate atherosclerosis, and demonstrates that therapeutic targeting of upregulated inflammatory pathways in patients with psoriasis leads to improvement and reduction in vascular diseases. Future studies should evaluate the potential use of dual antagonism of IFN-γ and TNF-α in reducing vascular diseases associated with psoriasis.

## Methods

### Study Approval

All protocols were approved by the institutional review boards (IRBs) of the University of Michigan, Ann Arbor, and the National Heart, Lung and Blood Institute. The study was done in accordance with the Declaration of Helsinki Principles and written informed consent was received from participants prior to inclusion in the study.

### Patient cohort

The study design included psoriatic and atherosclerotic global transcriptome data, along with histologic, qRT-PCR and *ex vivo* atherosclerotic and *in-vitro* stimulations of endothelial cells, as described below. Transcriptome data was obtained from patients with active chronic plaque psoriasis (n = 216 microarray and 12 RNA-seq) and skin from healthy controls (n = 6 RNA-seq), as described in our previous publications^48,49^. Transcriptomic data from early (n = 13) and late (n = 16) atherosclerotic carotid plaques were obtained from GEO Omnibus (https://www.ncbi.nlm.nih.gov/geo/) (GSE28829) as described in^12^. Arterial samples were obtained from discarded tissue from patients undergoing heart transplants (n = 8, 4 from patients with ischemic heart disease and marked atherosclerosis and 4 from patients with cardiomyopathy and no evidence of gross atherosclerosis on visual inspection, and/or elective amputations in patients with established peripheral atherosclerotic disease (n = 6). RNA-sequencing was performed on the coronary artery samples (n = 8), while samples obtained from amputations were used for *ex vivo* stimulations. Serum samples were collected from two separate cohorts of patients with active psoriasis (n = 120, n = 112) and healthy controls (n = 54, n = 29 (Suppl. Table 1). Exclusion criteria for participation included a history of systemic inflammatory disease other than psoriasis, known vascular disease, active infectious disease and uncontrolled hypertension. Patients without psoriasis were age and gender matched and derived from the same site as the psoriasis subjects from an ongoing healthy volunteer protocol.

### Bioinformatic analyses

Gene expression in lesional (PP) and non-lesional (PN) skin from psoriasis patients from 216 individuals (described in Swindell WR, *et al*.^50^) was analyzed using a commercial oligonucleotide microarray platform (Affymetrix Human Genome U133 Plus 2.0 Array)^50^. Likewise, this same microarray platform was used to compare expression between early (*n* = 13) and advanced (*n* = 16) atherosclerotic plaques (GSE28829)^12^. Bioinformatic analyses were performed as described (Supplementary File).

### Keratinocyte stimulation

Gene-set enrichment analyses to assess expression of psoriasis/atherosclerosis overlapping genes was done using a series of 42 microarray experiments in which cultured keratinocytes were treated with cytokines or cytokine combinations based on the cytokines up-regulated in psoriatic or atherosclerotic plaques^48^. Briefly, keratinocytes were grown to confluence and stimulated for 24 hours and RNA isolated and analyzed on an oligonucleotide platform as described^51^.

### Serum Analyses

Serum was obtained from two independent cohorts of patients 120 patients with moderate-to-severe psoriasis and 29 healthy non-psoriatic controls, and a separate cohort of 112 patients and 54 healthy controls (Supplemental Tables 1 and 2). Serum levels of IFN-γ, TNF-α, TNF-RI, and TNF-RII were measured from psoriasis or non-psoriasis controls via a Meso Scale Discovery 30-plex panel (MSD, Gaithersburg, MD).

### Arterial organ culture system, tissue processing, immunohistochemistry, ELISA

Sections of arterial tissue were obtained from vascular surgery, transferred to a sterile hood, and washed with HBSS (Invitrogen) to remove debris. The tissue sections were bisected longitudinally and then sliced into pieces approximately 2 mm thick. The arterial pieces were cultured on a 48-well plate in 300 ul per well of RPMI (Gibco) supplemented with Fetal Bovine Serum (Atlanta Biologicals) and Antibiotic-Antimycotic (Invitrogen) and stimulated with the following conditions: Un-stimulated control, interferon (IFN)-γ at 0.5 ng/ml, 5 ng/ml and 50 ng/ml (IFN-γ, R&D Systems), TNF-α at 1 ng/ml and 10 ng/ml (TNF-α, R&D Systems), and IFN-γ 50 ng/ml + TNF-a 10 ng/ml. Tissue was harvested after 6 hours for RNA, snap frozen in liquid nitrogen, and stored at −80 °C until RNA was extracted for qRT-PCR. The remaining duplicate pieces were harvested after 72-hour stimulation, fixed in 10% formalin overnight then stored in 70% ethanol for paraffin embedding. The supernatants of the 72-hour samples were collected, stored at −80 °C, until analysis with an enzyme-linked immunosorbent assay (ELISA). qRT-PCR was performed using the 7990HT Fast Real-Time PCR system (Applied Biosystems) with Taqman primers (Applied Biosystems, CXCL8, Hs00174103; CXCL9, Hs0017065; CXCL10, Hs01124251; RPLP0, Hs99999902). Immunhistochemistry was performed on 5 μm thick paraffin sections using antibodies against IFNGR1 (Fisher Scientific), IFNGR2 (Abnova Corp), TNFR1 (Lifespan Biosciences), and TNFR2 (Lifespan Biosciences). ELISA was performed for CXCL9 and CXCL10 (R&D Systems).

### Human aortic endothelial cell stimulation (HAoEC)

Primary human aortic endothelial cells (HAoEC, PromoCell, Germany) were cultured according to manufacturer’s recommendation using the corresponding media as well as the split kit (all, PromoCell, Germany). Cells were used up to passage 8. HAoECs were stimulated for 2 hours with 50 ng/ml IFN-γ, 10 ng/ml TNF-α, or 50 ng/ml IFN-γ + 10 ng/ml TNF-α. Subsequent to the 2-hour incubation, the HAoECs were harvested and RNA was extracted using a Directo-zol RNA MiniPrep (Zymo Research). The extracted RNA was converted to cDNA utilizing the Qiagen RT^2^ Strand Kit and gene expression was analyzed on custom RT-PCR plates for endothelial cell activation markers (Qiagen). Gene expression was reported as fold-change relative to untreated HAoECs.

### Impedance measurement of transendothelial electrical resistance

The electric cell substrate impedance-sensing technique (ECISZΘ, Applied BioPhysics Inc, Troy, NY, USA) was used to determine transendothelial electrical resistance (TER) as a measure of HAoEC barrier integrity. HAoEC were seeded into equilibrated ECIS arrays and grown to confluency for 5–7 days. TER was measured at 4,000 Hz for 1 h to allow stabilization of HAoECs in electrode arrays. Afterwards, cells were treated with 50 ng/ml IFN-γ, 10 ng/ml TNF-α, or 50 ng/ml IFN-γ + 10 ng/ml TNF-α. Changes in endothelial barrier integrity were observed by ongoing measurement of TER at 4000 Hz. Results were analyzed using ECIS, Excel and PRISM software. Experiments were carried out with n = 5.

### Human aortic endothelial cells immunofluorescence

HAoECs were grown to confluence for 5–7 days and treated with 50 ng/ml IFN-γ, 10 ng/ml TNF-α, or 50 ng/ml IFN-γ + 10 ng/ml TNF-α for 24 hours. HAoECs were fixed using 4% PFA/PBS (pH7.4) for 10 minutes at room temperature, permeabilized using 0.1% TritonX-100/PBS for 5 minutes at room temperature and blocked with 2% BSA/10%NGS in PBS (+Ca/Mg) for 30 minutes at room temperature. A primary antibody recognizing the adherence junction protein VE-cadherin was added over night at 4 °C (1:100) in 2% BSA/PBS (+Ca/Mg). Following 3 washes with PBS, cy3 labeled 2^nd^ antibody (1:300) and Phalloidin-Alexa488 (1:100, labeling of actin cytoskeleton) in 2% BSA/PBS (+Ca/Mg) were added for 1 hour at room temperature in the dark. Nuclei were labeled using DAPI and cells were mounted with DAKO fluorescence mounting media (DAKO). Fluorescently labeled cells were analyzed and photographed using an Axiovert (Zeiss, Germany) microscope. Immunofluorescence was performed in three independent experiment.

### Data and materials availability

Microarray data used in this manuscript are available on GEO (GSE28829, GSE40263, GSE13355, GSE14905, GSE30999).

## Electronic supplementary material

Supplementary Information
