# Supplementary material for: IFN-γ and TNF-α synergism may provide a link between psoriasis and inflammatory atherogenesis
Source: Sci Rep. 2017 Oct 23;7:13831. doi: 10.1038/s41598-017-14365-1 (PMC5653789; doi:10.1038/s41598-017-14365-1)
Supplement: Supplementary file 1 — Supplementary Information [file 41598_2017_14365_MOESM1_ESM.pdf]

SUPPLEMENTARY INFORMATION

IFN-γ and TNF-α synergism may provide a link between psoriasis and inflammatory atherogenesis.

Authors: Nehal N. Mehta[1], Heather L. Teague[1], William R. Swindell[2], Yvonne Baumer[1], Nicole L. Ward[3], Xianying Xing[2], Brooke Baugous[2], Andrew Johnston[2], Aditya A. Joshi[1], Joanna Silverman[1], Drew H. Barnes[2], Liza Wolterink[2], Rajan P. Nair[2], Philip E. Stuart[2], Martin Playford[1], John J. Voorhees[2], Mrinal K. Sarkar[2], James T. Elder[2], Katherine Gallagher[4], Santhi K. Ganesh[5], Johann E. Gudjonsson[2]

[1]National Heart Lung and Blood Institute, National Institutes of Health, Bethesda, MD
[2]Department of Dermatology, Univ. of Michigan, Ann Arbor, MI
[3]Department of Dermatology, Case Western Reserve University, Cleveland, OH
[4]Department of Surgery, Division of Vascular Surgery, Univ. of Michigan, Ann Arbor, MI
[5]Department of Internal Medicine, Division of Cardiovascular Medicine, and Department of Human Genetics, Univ. of Michigan, Ann Arbor, MI

Bioinformatic Analyses

Microarray datasets were normalized using Robust Multichip Average (RMA)[3]. Differential expression analyses were performed using linear models with moderated t-statistics (R package: limma)[4]. To control the false discovery (FDR) for multiple hypothesis testing, raw p-values from linear models were adjusted using the Benjamini-Hochberg method [5]. In psoriasis lesions, this analysis yielded 438 PP-increased DEGs (FC > 2.0 and FDR < 0.05) along with 196 PP-decreased DEGs (FC < 0.50 and FDR < 0.05). Using less stringent fold-change thresholds, we identified 1019 PP-increased DEGs (FC > 1.50 and FDR < 0.05) and 885 PP-decreased DEGs (FC < 0.67 and FDR < 0.05). Similarly, given the latter FC threshold of 1.50 (0.67), we identified 880 DEGs with increased expression in advanced atherosclerotic plaques, as well as 546 DEGs with decreased expression in advanced plaques (FC < 0.67 and FDR < 0.05). The 438 PP-increased DEGs (FC > 2) were examined in the atherosclerosis dataset to determine whether the proportion of genes with elevated expression in advanced plaques (FC > 1) was significantly large (as compared to all other skin-expressed genes; Fisher's Exact Test). The 196 PP-decreased DEGs (FC < 0.50) were similarly examined in the atherosclerosis dataset to determine whether the proportion of genes with decreased expression (FC < 1) in advanced plaques was significantly large (as compared to all other skin-expressed genes; Fisher's Exact Test).

We identified 115 DEGs increased in both psoriasis lesions and advanced atherosclerotic plaques (FC > 1.50 and FDR < 0.05 for both), along with 91 DEGs decreased in both psoriasis and advanced plaques (FC < 0.67 and FDR < 0.05 for both). The GOstats R package was used to identify Gene Ontology Biological Process terms significantly enriched with respect to these two gene sets, respectively[6]. Expression of these genes was further evaluated in 24 cell populations, based upon a database of 687 microarray samples described previously (Affymetrix Human Genome U133 Plus 2.0 Array)[7].

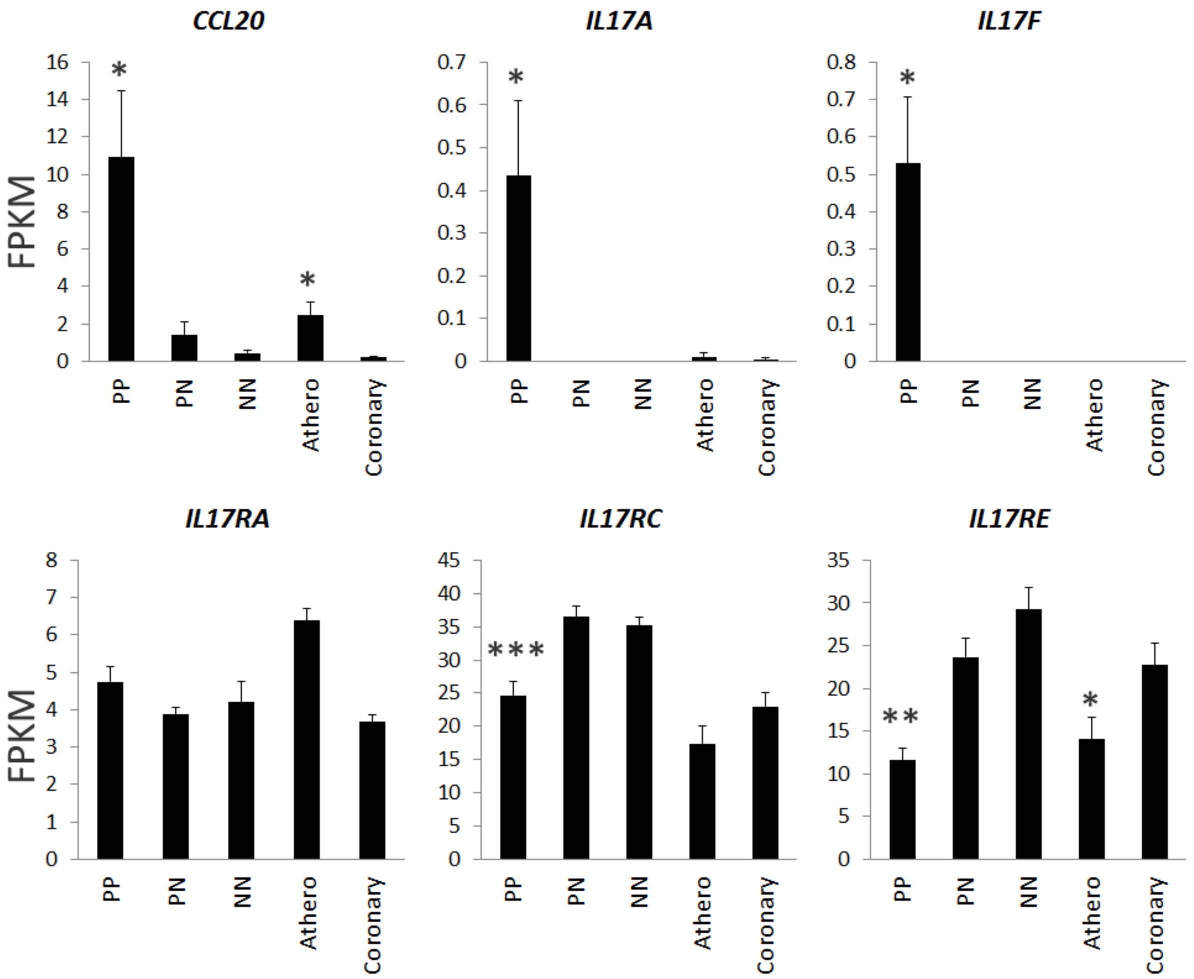

Supplemental Figure 1

Gene expression of IL-17 related cytokines (*IL17A*, *IL17F*), chemokines (*CCL20*), and receptors (*IL17RA*, *IL17RC*, *IL17RE*) in psoriatic (PP), uninvolved (PN), healthy (NN), atherosclerotic (athero) and healthy coronary vascular tissue (coronary). IL17A and IL17F had low to undetectable expression in both atherosclerotic and healthy coronary tissue while markedly elevated in psoriatic plaques. CCL20 was increased in both psoriatic plaque skin and atherosclerotic tissue. Expression of the IL17 receptors was detectable at very similar expression levels in all samples (vascular and skin) (n=6 lesional (PP), non-lesional (PN) and healthy (NN), n=4 atherosclerotic (athero), n=4 healthy vascular tissue (coronary), **, p<0.01, ***p<0.001, two-tailed Student's t-tests).

Supplemental Table 1: Baseline characteristics of psoriasis patients and healthy controls.

Parameter	Psoriasis (N = 120)	Controls (N = 29)	p-value
**Demographics and medical history**			
Age, years	47.9 ± 14.1	49.2 ± 12.9	0.33
Males	59 (49)	13 (45)	0.68
Hypertension	33 (28)	-	-
Type 2 diabetes mellitus	1 (1)	-	-
Current tobacco use	29 (24)	-	-
Lipid treatment	10 (8)	-	-
Body mass index, kg/m^2	29.3 ± 6.7	27.5 ± 6.0	0.09
**Clinical and laboratory values**			
Total cholesterol, mg/dL	195.0 ± 41.5	-	-
HDL cholesterol, mg/dL	63.7 ± 39.8	-	-
LDL cholesterol, mg/dL	111.9 ± 36.1	-	-
Triglycerides, mg/dL	133.5 ± 98.8	-	-
C-reactive protein, mg/L	1.9 (0.7 - 4.5)	-	-
**Psoriasis characterization**			
Psoriasis area severity index score	4.85 (2.35 – 10.08)	-	-
Systemic or biologic treatment	24 (20)	-	-

Values reported in the table as Mean ± SD or Median (IQR) for continuous data and N (%) for categorical data. P value less than 0.05 deemed significant. P values were calculated by using student's t-test or Mann-whitney U test for continuous variables and Pearson's chi-squared test for categorical variables.

Supplemental Table 2

	**PATIENTS (**N=112**)**	**CONTROLS (**N=54**)**
**AGE**	48.1 (range 16-83)	48,2 (range 18-83)
**FEMALE**	47.3%	48.2%
**BSA**	26,7% (range 0.1-87%)	0%

REFERENCES

1. Swindell WR, Stuart PE, Sarkar MK, Voorhees JJ, Elder JT, Johnston A and Gudjonsson JE. Cellular dissection of psoriasis for transcriptome analyses and the post-GWAS era. *BMC medical genomics*. 2014;7:27.

2. Doring Y, Manthey HD, Drechsler M, Lievens D, Megens RT, Soehnlein O, Busch M, Manca M, Koenen RR, Pelisek J, Daemen MJ, Lutgens E, Zenke M, Binder CJ, Weber C and Zernecke A. Auto-antigenic protein-DNA complexes stimulate plasmacytoid dendritic cells to promote atherosclerosis. *Circulation*. 2012;125:1673-83.

3. Irizarry RA, Bolstad BM, Collin F, Cope LM, Hobbs B and Speed TP. Summaries of Affymetrix GeneChip probe level data. *Nucleic acids research*. 2003;31:e15.

4. Smyth GK. Linear models and empirical bayes methods for assessing differential expression in microarray experiments. *Statistical applications in genetics and molecular biology*. 2004;3:Article3.

5. Benjamini Y and Hochberg Y. Controlling the False Discovery Rate - a Practical and Powerful Approach to Multiple Testing. *J Roy Stat Soc B Met*. 1995;57:289-300.

6. Falcon S and Gentleman R. Using GOstats to test gene lists for GO term association. *Bioinformatics*. 2007;23:257-258.

7. Swindell WR, Johnston A, Voorhees JJ, Elder JT and Gudjonsson JE. Dissecting the psoriasis transcriptome: inflammatory- and cytokine-driven gene expression in lesions from 163 patients. *BMC genomics*. 2013;14:527.
